# Generalization of Skill for a Working Memory Recognition Procedure in Children: The Benefit of Starting with Easy Materials

**DOI:** 10.3390/jintelligence11030056

**Published:** 2023-03-17

**Authors:** Chenye Bao, Nelson Cowan

**Affiliations:** Department of Psychological Sciences, Faculty of Arts and Science, University of Missouri, Columbia, MO 65211, USA

**Keywords:** working memory, practice, transfer effects, generalization

## Abstract

When children practice a new task, they need to learn both the task procedure and the materials tested. It is often unclear if improvements with practice reflect learning of the task procedure or familiarity with the materials. We sought to examine learning of the task procedure by switching from one set of materials to another in a working memory recognition task. We recruited 70 children (34 female, *M* = 11.27 years, *SD* = 0.62, ranging from 10.08 to 12.39) in the United States who were to remember sequences of orientations and of shapes for recognition immediately following the list. Half of the children began with orientation, an easier task, and the other half began with difficult-to-name shapes, a harder task. When children began with the easier task, the acquisition of the recognition task skill in the easy condition transferred to the more difficult task, optimizing the mean performance across tasks. Transfer was less potent when children began with the more difficult task. The results showed that sufficient practice is crucial to avoid poor initial performance, which might be important for the student’s rate of progress and task engagement.

## 1. Introduction

When children are asked to carry out a laboratory task, their performance depends upon both familiarity with the material and practice with the task with which it is typically tested. It is typically difficult to know how much learning consists of familiarity with the materials and how much consists of practice with the task. We investigated this issue in elementary school children by changing the materials but using the same working memory recognition task for two sets of materials in sequence. Any transfer of learning of the task would result in better performance for the first part of the second set of materials, compared with the performance for the first part of the first set of materials.

Working memory refers to a system that provides the temporary storage and manipulation of information that is necessary for engaging in a wide range of complex activities (Baddeley 2012). It is generally regarded as a limited capacity to maintain only a relatively small number of items, with a capacity that varies among individuals (Baddeley 2012; Cowan 2001). An individual’s capacity, and the manageability of the particular materials to be remembered given that capacity, is important for learning and cognitive performance of many kinds. For example, Cowan (2014) summarized evidence that working memory capacity helps determine how well information can be learned, inasmuch as learning involves holding the combination of various pieces of information in the focus of attention at once so that new associations can be formed. Relevant information must be held in mind while a process is carried out. Presumably, for that reason, working memory is highly correlated with success in a broad range of cognitive activities, including as language comprehension (Daneman and Merikle 1996), reading (Peng et al. 2018), problem-solving and arithmetic (Swanson and Beebe-Frankenberger 2004), and decision-making (Hoffmann et al. 2020).

Here, in a version of a short-term recognition task, we disentangled two factors that were confounded in past work, both of which are potentially important in influencing performance, namely familiarity with the materials to be learned, on the one hand, and practice with the task, on the other hand. Either factor could underlie the effects of practice that have been observed previously (e.g., Chen et al. 2006; Morrison and Chein 2011; Rudebeck et al. 2012). We separated the factors by having the participants practice a short-term recognition task, first with one set of materials, either line orientations or shapes, and then with another set, then finally returning to the first set. The issue was whether practicing this task with one set of materials would enhance performance when the participant switched to the other set. We first reviewed prior research on working memory and task familiarity and explain here how we went beyond this literature.

## 2. Working Memory and Task Familiarity

It is clear that familiarity with a stimulus increases performance in short-term recognition tasks. (e.g., Curby and Gauthier 2007; Morrison and Chein 2011). For example, Buttle and Raymond (2003) presented a pair of faces followed by a second pair, with one face the same and the other changed to a different individual of the same gender. Recognition was superior when the change involved a famous face as the pre-change face, the post-change face, or both. However, this effect occurred only when the change was on the left side of the screen. This effect of face familiarity was eliminated when the faces were inverted, which suggested that it required facial recognition. Others, however, have not found a beneficial effect of familiarity. Chen et al. (2006) compared short-term recognition of novel and trained polygons. The results show that performance improved during practice, but that this improvement was not limited to polygons used in training. These findings indicated that familiarity with unnamable shapes played a limited role in influencing the ability of visual working memory.

Various studies have been conducted in recent years under the heading of working memory training, but these studies mostly have examined effects of practice, not the introduction of a specific strategy for the participants to use. A growing number of working memory training studies have explored the effects of working memory training on diverse cognitive abilities in different populations (Shipstead et al. 2012; Sternberg 2008). However, the current conclusions regarding working memory training are inconsistent (Chen et al. 2006; Corbin and Camos 2011; Henry et al. 2014; Holmes et al. 2009; Klingberg et al. 2002; Melby-Lervåg and Hulme 2013; Melby-Lervåg et al. 2016; Stephenson and Halpern 2013). Claims have been made that working memory training can improve intelligence (Klingberg et al. 2002), reading comprehension (Dahlin 2011), mathematical ability (Holmes et al. 2009), and even future scholastic success (Alloway 2009; Cowan et al. 2005). “Transfer of training” to other tasks or situations (for example, training on a working memory task leading to better performance on scholastic tasks) is a critical indicator of working memory training used to evaluate the enhancement and efficiency of processing through training (Barnett and Ceci 2002). It is quite common in working memory training literature to equate post-test improvements in a trained task to improvements in cognitive ability (Jaeggi et al. 2008; Klingberg 2010). However, better performance on a particular task does not signal an improvement in working memory’s capacity per se (Shipstead et al. 2012). The literature has mostly shown that it is possible to become better with practice and that it is possible to show the transfer of skills from one task to a similar task, called near transfer (Shipstead et al. 2010). For example, improvements on simple-span tasks following training on an adaptive n-back task can be considered a near-transfer effect. There is little evidence of “far transfer,” that is, improvement in tasks very unlike the working memory tasks that were used for training, such as fluid intelligence, reasoning, nonverbal ability, verbal ability, arithmetic, word decoding, or reading comprehension (for reviews, see Shipstead et al. 2012; Melby-Lervåg et al. 2016).

What is known about the near transfer of training within working memory studies does not completely distinguish between learning of the task procedures and familiarity with the materials to be learned. Melby-Lervåg et al. (2016) conducted a meta-analysis study involving 87 working memory training studies and found no far-transfer effects but found reliable near-transfer effects. A few studies explored the potentially domain-specific nature of working memory training effects, the tendency for training to apply to one particular domain, such as transference from one visual memory task to another, but not another domain, such as transference from visual to verbal memory (e.g., Melby-Lervåg and Hulme 2013; Von Bastian and Oberauer 2014). A recent empirical study also focused on domain-specific transfer effects (Stephenson and Halpern 2013), reporting transfer effects for matrix reasoning following visual-spatial working memory training but not following auditory n-back training. However, even the extent of within-domain transfer is uncertain.

## 3. The Present Study

Within the near transfer that occurs, it is not clear whether the transferred benefit comes from similarities in the materials (e.g., transferring from a task using digits to a task using letters as the memoranda) or similarities in the task (e.g., transferring from recognizing to recalling digits). We believe that one way to begin to assess the role of task practice with a transfer of the training procedure is to keep the task’s requirements fixed and change only the materials to be remembered from one type to a different type, within that task. We distinguished between these possibilities by using the same short-term recognition procedure with two kinds of materials, line orientations and shapes, with a switch from one type to the other and back again for a single participant.

**Figure 1:**
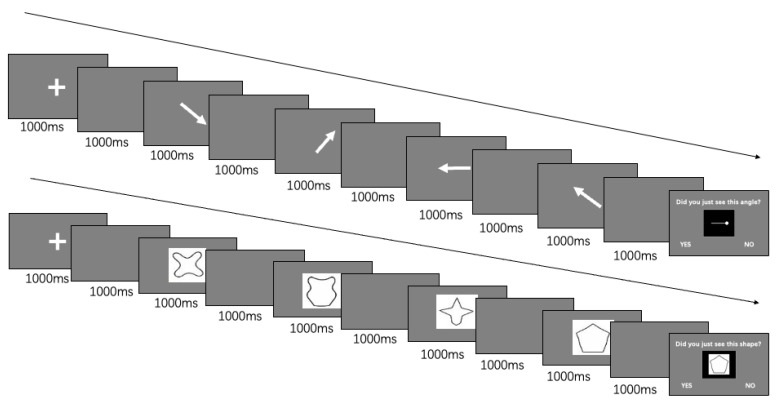
Detailed illustration of an orientation and a shape trial. Note. The upper row was an orientation test trial; the lower one was a shape test trial.

The purpose of the present work was to document the role of task practice within the near-training effects that have been examined. We did this by changing the materials to be remembered while keeping the requirements of the task fixed. The basic procedure is illustrated in Figure 1. Items were presented one at a time in a sequence, each in the middle of the viewing screen, followed by a test of the recognition of one item, which was to be judged to have been included in the most recent sequence or absent from it. After an initial block of trials with one type of item (line orientations or unfamiliar shapes), the same type was used in a second block that began with the open-ended encouragement to remember the items through whatever strategy worked best. This was followed by two blocks of trials in which the second type of items was used instead: shapes if orientations had been presented first, or orientations if shapes had been presented first. Finally, there was a trial block in which the initial type of items was presented once more. Testing each new type of item introduced began with practice trials that were not counted. The main predictions were (1) that the performance at the beginning of the materials tested second would be at a higher level than at the beginning of the materials tested first, due to learning of the recognition test procedure; and (2) that the performance at the return to the initial test procedure in the final block would be at the same practiced level as when it was last tested.

In the process of pilot testing, we learned that recognition of unfamiliar shapes was considerably more difficult than recognition of line orientations. This observation led us to the eventual realization that learning the recognition procedure might be facilitated by starting with the easier line-orientation task.

Given the relevance of our investigation for classroom learning, we decided to examine performance in 10- and 12-year-old children, who seemed old enough to carry out the recognition procedure according to instructions and benefit from practice, but young enough to have a simple memory span approaching adult-like levels (e.g., Cowan et al. 1987).

## 4. Material and Methods

### 4.1. Participants

We recruited 70 children (34 female and 36 male, *M* = 11.27 years, *SD* = 0.62, ranging from 10.08 to 12.39 years) from primary schools in the United States. They were randomly assigned into two groups, which differed in the order in which two types of stimuli were presented to each group. Group One had 14 females and 21 males, *M* = 11.18 years, *SD* = 0.61, ranging from 10.11 to 12.35 years; Group Two had 20 females and 15 males, *M* = 11.35 years, *SD* = 0.62, ranging from 10.08 to 12.39 years. Parents were encouraged to complete the electronic consent and demographic information before children participated in the online experiment. The sample was composed of approximately 72.86% White children, 15.71% Asian-American children, 7.14% African-American children, 1.43% children of other races, and 2.86% of participants preferring not to say according to the parents’ self-reported records. The study was approved by the institutional review board of the University of Missouri.

Our analyses of the data are based on Bayesian inferential statistics (e.g., Rouder et al. 2012). These statistics yield evidence that can provide positive support either for or against the existence of an effect. Increasing the sample size offers more certainty about the correct interpretation of the findings but, unlike frequentist analyses, it does not run the risk of eventually finding a significant effect that is too small to be of practical significance; instead, the existence versus nonexistence of each effect becomes clearer with an increasing N. As is common using Bayesian methods, therefore, we allowed ourselves to increase the sample size past an initial minimum size of 40, subsequently increasing it to 70 so that the results were no longer in an indeterminate range.

### 4.2. Design

There were seven phases of the experiment, each of which involved trials with a brief presentation of a four-object sequence followed by a probe to be recognized as present in the sequence or absent from it. The phases were: (1) practicing Task 1 (Practice1), (2) testing on Task 1 (Stim1Part1), (3) further testing on Task 1 (Stim1Part2), (4) practicing Task 2 (Practice2), (5) testing on Task 2 (Stim2Part1), (6) further testing on Task 2 (Stim2Part2), and (7) further testing on Task 1 (Stim1Return). For Group 1, Task 1 involved sequences of identical objects with different orientations and Task 2 involved sequences of objects of different shapes, whereas for Group 2, Task 1 used shapes and Task 2 used orientations. There were 4 practice trials in each practice phase and 20 trials in each test phase, 10 with the probe present in the sequence and 10 with the probe absent from the sequence. Before Stim1Part2 and Stim2Part2, the second phase for each stimulus type, there were instructions for the participants to try to find the best way to remember the stimuli. Figure 1 illustrates one orientation trial and one shape trial.

### 4.3. Stimuli and Procedure

Each participant was tested individually in a quiet room via a popular program allowing interactions between the experimenter and the participant over the computer by audio and video, ZOOM. An experimenter was present throughout the whole experiment for the children online. The experimental program was created and run in PsychoPy v2021.2.3. The researcher ran the experimental program on her own computer and shared the screen with one participant at a time. Participants were allowed to use a desktop computer, laptop, or iPad to carry out the tasks. Before starting the experiment, an experimenter explained the purpose of the present study and the instructions for each phase. Then there was an opportunity to ask questions, and the participant was encouraged to complete the experiment. They were told that they could stop the experiment at any time if they felt uncomfortable. No participant asked to stop the experiment. Lastly, an electronic payment form was to be completed by their parents after the online session. They received $15 for completing the whole experiment, which lasted about 35–50 min.

For Group 1, the first phase was practicing with the orientation stimuli. Only one orientation item was presented at the center of the screen in the first trial. Then the second practice trial presented two orientation stimuli in sequence, then three, and finally four, summing to four practice trials in total. In each trial, participants were asked whether they had seen a probe stimulus or not within the just-seen sequence. The options “Yes” and “No” were in the lower left part and the lower right part of the screen, respectively. Children spoke their responses, and then the researcher clicked their answers immediately. In the practice phases, the children were required to correctly answer each time before starting the next trial to ensure they understood the experiment.

Children received feedback after they selected the answer in the practice phase. Then the test trials were presented, each with a four-object visual sequence followed by a single probe object, to be judged as the same as one of the four sequence objects or different from all four (in either shape or orientation, depending on the current task). They did not receive feedback during the test phases.

In Group 1, the test phases Stim1Part1 and Stim1Part2 had 20 four-item trials each, with orientation stimuli. Importantly, the instructions to find the best way to remember these stimuli were given to participants at the beginning of Stim1Part2. Then the participant switched to the second set of stimuli, shape stimuli. Practice2 included four practice trials, and Stim2Part1 and Stim2Part2 were test trials, with four-item shape stimulus sequences. The instructions to find the best way to remember these stimuli were presented at the beginning of Stim2Part2. Finally, the test phase Stim1Return was again the orientation stimulus with four-item sequences. Across the test phases, there were 100 test trials (60 trials for the first set of stimuli and 40 trials for the second set of stimuli) and 8 practical trials.

Group 2 followed the same procedure as Group 1, but the stimulus types used were the opposite. Specifically, Group 2’s participants were presented with the sets of shape stimuli in Practice1, Stim1Part1, Stim1Part2, and Stim1Return, and with orientation stimuli in Practice2, Stim2Part1, and Stim2Part2.

As shown in Figure 1, all items were presented on a screen with a uniform medium gray background. A trial began with a 1000 ms fixation cross at the center of the screen, followed by a 1000 ms blank interval. Then came the four different items to be remembered in sequence, each lasting 1000 ms. The items were separated by an interstimulus interval (ISI) of 1000 ms. The test item was presented last and remained on the screen until a response was recorded. Additionally, the practice trials displayed feedback reporting correct or incorrect responses and instructing the participant to restart the trial or begin the next trial.

As Figure 1 (top row) shows, the orientation items presented in each trial were arranged in eight different directions (north, south, east, west, northeast, northwest, southeast, and southwest). The figure used for the orientation test was an arrow presented in white on a gray background and, when viewed horizontally, took up 21% of the screen’s width (on the experimenter’s screen, 545 of 2560 pixels in width, scaled up or down to equal the same proportion on the participant’s screen). It was rotated around the center of the figure, and four different orientations were shown in each test sequence to be remembered. The probe to be identified as an orientation present in the series or absent from it was shown as a white pin within a black square taking up 21% of the width of the screen (on the experimenter’s screen, 545 of 2560 pixels in width and 545 of 1600 pixels in height), to distinguish the probe from the memoranda.

As shown in Figure 1 (bottom row), the shape items included eight different irregular shapes, which were developed by Li et al. (2020). Each shape was centered on the screen and was presented in black (Figure 1), filling most of a white square that covered 21% of the screen’s width. After four stimuli were presented, the test item was shown with a black border around the white background to distinguish the test item from the other stimuli (Figure 1). For the serial presentation of lists of objects for an immediate recognition task, a previous study showed that preventing articulatory activity upon the presentation of each object did not change the pattern of the results (Cowan et al. 2011), so we did not add that complication to the present study.

### 4.4. Statistical Analysis

In the present study, the software package R Studio was used for descriptive statistical analyses for the proportion of responses correct in the two groups in different test phases. We relied on Bayesian tests (ANOVAs and *t*-tests) for inferential purposes but we also provide frequentist results, for descriptive purposes. We conducted a Bayesian analysis, which is a statistical inference method that calculates the ratio of the likelihood of each model including an effect to the likelihood of the identical model excluding that effect. The Bayesian analyses used the Cauchy prior distributed as $\pi \left(\mu ,\;\sigma^{2}\right)=\frac{1}{\sigma^{2}}$ (Rouder et al. 2012). The R package we used assumed a Cauchy distribution of effect sizes centered on 0, with a scale of 0.707 (Ly et al. 2018). The resulting Bayes factor for the inclusion of the effect (BF_incl_) of no less than 3 is usually considered evidence for an effect, while a BF less than 0.33 is considered to be evidence against the effect, with more extreme values indicating stronger evidence.

## 5. Results

We examined three dependent variables: the proportion of correct recognition, which indicated how well the stimuli were remembered; the reaction time, as reported in the online supplement; and bias, a measure of an individual’s overall tendency to say that a probe was or was not included in the studied sequence. For all measures, we excluded trials with reaction times exceeding the overall mean plus 3 standard deviations, which were trials with reaction times of 13.81 s or above. Out of 7000 test trials, 78 trials (1.11%) were omitted on the basis of this criterion. The averages of the proportion correct and the reaction time in each test phase for the two groups are shown in Table 1.

### 5.1. Proportion of Correct Recognition

**Figure 2:**
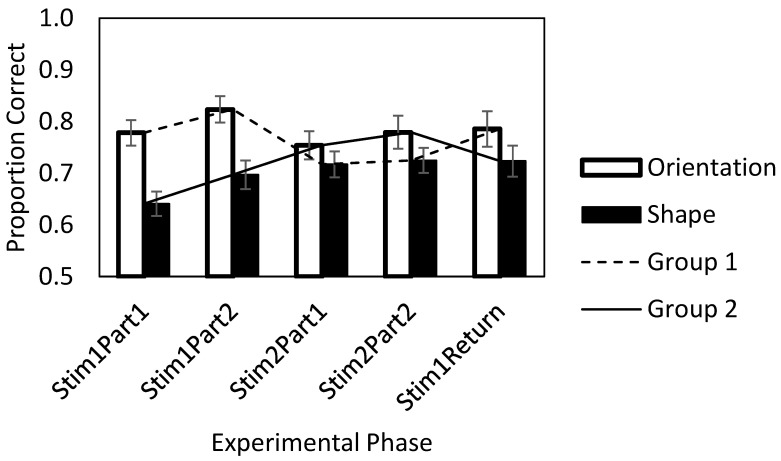
The proportion correct in each phase (x-axis) of each group. Note. Dashed line: Group 1 started with the orientation stimuli. Solid line: Group 2 started with the shape stimuli. Practice phases for the two stimuli were omitted from the figure. The error bars are the standard errors of the mean.

Figure 2 illustrates the mean proportion correct for five test phases for both groups. The patterns for the two groups in these five test phases were different. One can see that the performance for Group 2 was initially low for the shape trials and that the performance was higher in Group 2 later on for both kinds of stimuli. One can see that the performance began higher in Group 1 and that the switch to shape trials in this group in Stim2Part1 and Stim2Part2 did not result in the same poor performance that Group 2 initially showed. That is to say, the kind of stimulus interacted with the order in which the stimuli were presented. This description was confirmed with the statistical analyses of the proportion correct, as reported below.

The ANOVA of the proportion of correct recognition was conducted with one within-participant factor (phase, with five phases) and one between-participants factor (Group, 1 or 2). We examined seven models by comparing them with a null hypothesis model that did not include group, phase, or their interaction. The models, summarized in Table 2, included those with all combinations of the group and phase variables. On the basis of the table, we used three different methods to calculate BF_incl_ (the Bayes factor for including an effect) for each effect: phase, group, and their interaction (Table 3). In the first method, we compared the factor with the null model. For example, for the interaction effect, the Bayes factor of Model 4, which only included the interaction effect, indicated BF_incl_ = 268,376,003. Then the second method was to divide the effects that appeared to exist alone plus the tested effect by the effects that appeared to exist, without the tested effect. For the effect of the interaction, we divided the BF of the group model plus the interaction by the BF of the group model, that is, BF_incl_ = 238,534,312,357/796.57 = 299,453,294. Given that the Bayes factor for phase was small, we did not use it in a similar calculation to assess the interaction. Third, we compared the full model to a model omitting only the effect in question. For the interaction, we divided the full model by the model with both main effects but no interaction, so BF_incl_ = BF_Model 7_/BF_Model 3_ = 2,686,584,969/8.67 = 310,050,198. Overall, these three methods of calculating BF_incl_ produced quite similar results. We also used these three methods for calculating the BF_incl_ for the main effects of phase and group. When all three methods were used, the BF_incl_ of the effect of group was close to 800. However, the BF_incl_ of the effect of phase was about 0.01, as Table 3 shows. In summary, the best model in the present study was Model 6, which included the group and interaction variables but not the phase variable.

A three-way Bayesian ANOVA analysis that included not only the phase and group but also the participants’ gender did not show any reliable effects of gender (main effect, BF_incl_ = 0.48; interaction with group, BF_incl_ = 0.51; interaction with phase, BF_incl_ = 0.08; three-way interaction, BF_incl_ = 0.09). The first two effects were indeterminate, whereas the last two effects were small enough (<0.33) to provide positive evidence of a null effect, indicating that there was no gender difference in the learning process across phases.

To learn more about the patterns of the results, Bayesian t-tests were conducted for pairwise comparisons of the five test phases in each group (Table 4). Because each t-test yielded a Bayes factor that expresses a ratio that can either support the alternative hypothesis or support the null (or can be indeterminate), there was no bias toward finding an effect with more tests, and we believed that no correction for multiple testing would be appropriate. Table 4 shows the results of these comparisons. Within a group, most of the reliable differences occurred when the stimulus has changed from orientations to shapes or vice versa, indicating that orientations were generally easier than shapes. One exception was that, in Group 2, the performance on shapes improved from Phase 2 (Stim1Part1) to Phase 7 (Stim1Return), BF_incl_ = 12.39, a practice effect. We also carried out pairwise comparisons of the test phases that used the same stimulus across two groups (Table 4, bolded numbers). First, we compared the mean proportion correct for orientation as the first and the second stimulus (Stim1Part1 in Group 1 vs. Stim2Part1 in Group 2; Stim1Part2 in Group 1 vs. Stim2Part2 in Group 2). The results did not show reliable differences, indicating that the order in which the orientation stimuli were presented did not affect the children’s proportion of correct recognition. Second, we carried out similar comparisons for the mean proportion correct for shape (Stim1Part1 in Group 2 vs. Stim2Part1 in Group 1; Stim1Part2 in Group 2 vs. Stim2Part2 in Group 1). The results indicated only one reliable difference: the mean proportion correct for shape in Stim1Part1 in Group 2 was reliably lower than the one for shape in Stim2Part1 in Group 1 (BF_incl_ = 3.44), indicating that practice with the easier orientation stimuli transferred, to some extent, to the more difficult shape stimuli (see Figure 2).

### 5.2. Reaction Times

As shown in Table 1 and in the online supplement, starting with the easier orientation task in Group 1 resulted in a faster response throughout the entire experiment (*M* = 3.50 s) than starting with the more difficult shape task in Group 2 (*M* = 3.65 s). This finding was supported by the ANOVA analysis of reaction times (see the online supplement), BF_incl_ = 6.00 to 6.33 depending on the method of analysis. This difference in speed between the groups persisted even when the tasks were switched, so there was no interaction of group and the phase of the experiment (BFincl = 0.0005, or 2000 to 1 in favor of the null hypothesis). This may be related to the performance advantage of Group 1 conferred by starting with an easier stimulus (orientation) first. More details of the analysis and the results for the reaction time and bias are provided in the Supplementary Materials.

## 6. Discussion

The main finding of this study was that children showed near transfer from one task to another in a working memory training session. Children performed better on the more difficult task when they started to learn the easier materials first. Transfer effects of this sort are crucial for demonstrating the practical or clinical benefits of working memory training (Melby-Lervåg and Hulme 2013). Below, we summarize the results and then discuss the aspects of task difficulty, the equivalence of the initial ability, practice, and bias before examining the limitations of the study and prospects for future research.

By comparing the training performance of the two groups of participants, the findings of the Bayesian t-test comparison imply that if the training starts at a different level of difficulty, then it may affect the participants’ subsequent proportion correct during the training. Specifically, Group 1 began with the easier stimuli (orientation) before tackling the more difficult shape stimuli, whereas Group 2 began with the opposite, that is, they began with the more difficult stimuli (shapes) first and then switched to orientations. We found better performance for the more difficult materials in the short-term shape recognition task for the group that received these materials only after having practiced the short-term orientation recognition task. Specifically, Group 1′s first test performance levels with the difficult task in Phase 5 were much higher than Group 2′s first performance levels with the difficult task in Phase 2 (see Figure 2), which was a reliable difference (BF_incl_ = 3.44). There was no comparable transfer for initial training on the more difficult task; the switch from shape to orientation recognition in Group 2 produced a performance in Phase 5 that was no better than the performance of Group 1 in Phase 2 regarding orientation. The short-term orientation recognition task, which involved easier materials, yielded approximately equivalent performance in both groups; for the initial phase in each group, BF_incl_ = 0.09 or 11.11 to 1 (1/.09) in favor of the null hypothesis (see Table 4, first column).

On the basis of these findings, we suggest that the order of presentation of the stimuli may influence children’s proportion of correct recognition at the beginning of training. The advantage of starting with the easier stimuli is that the recognition procedure is already familiar by the time the more difficult shape stimuli are introduced. In summary, we found asymmetrical effects of working memory training on the transfer of training in a relatively short working memory training period for the first time in the literature, to our knowledge.

The above conclusions depend on the fact that the initial ability levels of the participants in both groups were equivalent prior to working memory training. In the current study, we did not perform an initial measurement of each participant’s ability, but when the participants had practiced, the scores of the groups were almost identical across the last two test phases (one orientation and one shape trial phase in each participant, in the opposite order (see Figure 2, Stim2Part2 and Stim1Return data). This equivalence suggests that the ability levels of the two groups were comparable and that the initial differences in performance within the groups were related to the material presented when it was unpracticed. Thus, we can conclude that the order of presentation influenced performance and that the learning procedure with more success along the way was the one starting with the easier materials.

The reason that starting with easier materials is helpful could be that children’s working memory is improved by the application of metamemory or insight into the capabilities of one’s own working memory (Forsberg et al. 2021), but this metamemory consumes some of the same resources as working memory storage. Metamemory, or knowledge of one’s own memory, can be used to fine-tune performance. For example, if you know that you can only hold three items of a certain kind, it is counterproductive to try to hold four, because that attempt will make you lose more of them (similar to dropping a too-tall stack of dishes). A more difficult set of materials (shapes compared with line orientations) leaves less of working memory’s capacity to be devoted to using metamemory to maximize performance.

This pattern of results underscores the potentially critical role of the amount of practice when comparing different experimental conditions in children. Moreover, starting with the easier orientation task in Group 1 resulted in faster responses throughout the entire experiment than starting with the more difficult shape task in Group 2. These findings suggest that by starting with simple tasks, children learn to respond more efficiently than when starting with more difficult tasks. In our procedure, the order of presentation ultimately did not matter for the final performance when there had been sufficient practice. However, in educational settings, it can be helpful to set up an experience of success rather than an initial experience of failure, which is an argument for starting with easier materials. Moreover, some learning situations do not afford enough practice to result in the final equivalent levels that we observed.

This type of working memory experience we have examined might be applicable to children’s development in other cognitive abilities and academic achievement, such as arithmetic and writing. Given that, throughout childhood, the contents of working memory are related to how much information children will transfer to their long-term memory (Forsberg et al. 2022), we expect that our findings are relevant to learning. For example, on the basis of the findings of this study, we hypothesize that children may learn to memorize their arithmetic multiplication tables more efficiently if they start by memorizing the lower part of the table soundly before problems with higher numbers are presented. Our finding can also be of use in fine-tuning studies of near transfer so they can be conducted in a way that distinguishes whether it is the materials or the task skill that is trained, or some of both.

## 7. Limitations and Prospects for Research

To our knowledge, our study is the first to find a near-transfer effect for working memory training (or practice) in a single session lasting less than an hour. Our study also had some limitations that need to be considered. First, because our training period was relatively short, between approximately 30–40 min, this may have result in training effects that did not last long. Therefore, future studies could conduct longer training periods to further explore the transfer effects (e.g., in longitudinal research). In addition, some past working memory training studies have found that working memory training improves individuals’ cognitive abilities (e.g., Jaeggi et al. 2008; Klingberg 2010), whereas other studies have not found this (e.g., Melby-Lervåg et al. 2016). Our training studies did not measure the children’s cognitive abilities before and after training. Although it seems unlikely that any such transfer would emerge in a short period, it might be helpful to check this, inasmuch as a positive result could reveal aspects of apparent far transfer that might be due to the transient situation, rather than due to true long-term improvement. Importantly, to reiterate the purpose and meaning of our working memory training, the goal was not to make individuals expert performers of specific tasks, but to explore the positive effects of rapid working memory training (or practice) that may indicate previously overlooked capabilities that are relevant to children’s development and, possibly, more generally to people’s daily lives (Chase and Ericsson 1982).

In our study, we trained children on one visual working memory task and transferred them to another visual memory task, but it is possible that there were skills needed in these tasks that would apply to other tasks, such as verbal working memory recognition tasks. The domain-generality of the finding has not been explored yet. Plenty of existing studies have demonstrated that working memory’s capability affects a variety of individuals’ cognitive abilities such as general fluid intelligence (Kane et al. 2005; Oberauer et al. 2005), reading comprehension (Daneman and Carpenter 1980; Turner and Engle 1989), reasoning (Kyllonen and Christal 1990; Von Bastian and Oberauer 2013), learning computer languages (Shute 1991), and academic achievement (Alloway 2009). One factor that might lead to the inconsistent results of working memory training studies is the nature of the training task. This argument reflects the intense debate over two competing working memory models: domain-general versus domain-specific (Shah and Miyake 1996). There is an ongoing debate on whether working memory’s capacity is a domain-general construct or a separable, domain-specific construct (Melby-Lervåg and Hulme 2013The domain-general perspective of working memory involves processes that are not related to specific types of information or sensory modalities but which still help to encode, maintain, and retrieve information from working memory. It is meant to apply to any area (Morrison and Chein 2011). Morrison and Chein thought that domain-general processes of working memory involved mechanisms that control attention, gate information flowing into and out of the working memory buffer in our brain, avoid interference from unnecessary information sources, and manage engagement with domain-specific strategies. Therefore, the type of stimulus, such as verbal or visual-spatial, used for working memory training should not affect the effect of training (Peng and Fuchs 2017). Others take a different view, namely the domain-specific perspective that working memory involves strategies that are specific to the maintenance and operation of specific types of information. Children’s studies tend to favor this point of view. For instance, children who participate in visual-spatial working memory training tasks would perform better in visual-spatial working memory and visual-spatial related tasks (e.g., arithmetic calculation) than verbal working memory tasks (Peng and Fuchs 2017). In clinical research, Swanson et al. (2009) thought that children with serious learning difficulties exhibited working memory deficits in both the verbal and visual-spatial domains. However, verbal working memory deficits are more related to reading difficulties in children, whereas visual-spatial deficits tend to be more important for difficulties with mathematics (Swanson and Jerman 2006). To expand the applicability of our findings, it would therefore be useful to examine whether domain-general skills are learned that would apply when there is a switch from visual to verbal working memory recognition tasks or vice versa, and from sequence item recognition tasks to list item recognition tasks or vice versa.

## 8. Conclusions

The findings of the current working memory training (or practice) study showed that children were able to show near transfer from one task to another after a working memory training session, with an advantage of starting with the easier task. The difficulty of tasks affected the children’s performance at the beginning of training. Moreover, starting with the easier task resulted in faster responses throughout the entire experiment than starting with the more difficult task. After some training (experience with the task), children’s average proportion correct tended to be similar across groups, which underscores the potentially critical role of the amount of practice when comparing different experimental conditions in children. Children may develop strategies on their own with practice during the training process. These findings may shed some light on children’s cognitive development.

## Figures and Tables

**Table 1:** The descriptive statistics of the proportion correct and reaction time for each phase.

Group	N	Phase	Stimulus	Proportion Correct		Reaction Time	
				Mean	SD	Mean	SD
1	35	Stim1Part1	Orientation	0.78	0.42	3.47	1.75
		Stim1Part2	Orientation	0.82	0.38	3.37	1.73
		Stim2Part1	Shape	0.72	0.45	3.57	1.80
		Stim2Part2	Shape	0.73	0.45	3.55	1.86
		Stim1Return	Orientation	0.79	0.41	3.56	2.13
Average				0.77	0.42	3.50	1.86
2	35	Stim1Part1	Shape	0.64	0.48	3.57	2.00
		Stim1Part2	Shape	0.70	0.46	3.45	1.76
		Stim2Part1	Orientation	0.75	0.43	3.83	1.88
		Stim2Part2	Orientation	0.78	0.42	3.79	1.92
		Stim1Return	Shape	0.72	0.45	3.62	1.93
Average				0.72	0.45	3.65	1.90
Grand average				0.74	0.44	3.58	1.88

**Table 2:** Statistical results from frequentist and Bayesian ANOVA for the proportion correct.

Model	Factors	Predictor	*df*		F	Cohen’s f	*p*	BF_incl_ of Model
1	Phase	Phase	4	276	1.97	0.046	0.10	0.01
2	Group	Group	1	6920	20.65	0.055	**<0.001**	796.57
3	Phase + Group	Phase	4	276	1.97	0.046	0.10	8.67
		Group	1	64	3.55	0.071	0.06	
4	Interaction	Interaction	8	272	6.38	0.110	**<0.001**	268,376,003
5	Phase + interaction	Phase	4	272	2.24	0.046	0.07	3,036,658
		Interaction	4	272	10.51	0.100	**<0.001**	
6	Group + interaction	Group	1	60	2.39	0.059	0.13	238,534,312,357
		Interaction	8	272	6.38	0.110	**<0.001**	
7	Phase + Group + interaction	Phase	4	272	2.24	0.046	0.07	2,686,584,969
		Group	1	60	3.49	0.071	0.07	
		Interaction	4	272	10.51	0.100	**<0.001**	

Note. The BF_incl_ of each model was in comparison with the null model.

**Table 3:** Three methods for calculating the Bayes factor for the proportion correct.

Factor	Method	Formula	BF_incl_
Phase	1	Phase/null model	0.01
	2	(Phase + Group)/Group	0.01
	3	All effects/(Group + interaction)	0.01
Group	1	Group/null model	796.57
	2	(Group + interaction)/interaction	888.81
	3	All effects/(Phase + interaction)	884.72
Interaction	1	Interaction/null model	268,376,003
	2	(Group + interaction)/Group	299,453,294
	3	All effects/(Group + Phase)	310,050,198

**Table 4:** Bayes factors for t-test comparisons of the proportion of correct recognition for comparable conditions.

	G1P2	G1P3	G1P5	G1P6	G1P7	G2P2	G2P3	G2P5	G2P6
G1P2	-								
G1P3	0.59	-							
G1P5	1.33	8263.23	-						
G1P6	0.34	1201.70	0.05	-					
G1P7	0.05	0.21	6.18	1.79	-				
G2P2	-	-	**3.44**	-	-	-			
G2P3	-	-	-	**0.14**	-	0.44	-		
G2P5	**0.09**	-	-	-	-	2103.25	0.84	-	
G2P6	-	**0.50**	-	-	-	771,403.70	31.13	0.08	-
G2P7	-	-	-	-	-	12.39	0.09	0.09	0.88

Note. Conditions selected for comparison included all those within the same group as well as cross-group comparisons of the first two phases for each type of stimulus. G1P2 = Group 1, Phase 2, and so on. Group 1 began with orientation stimuli and Group 2 began with shape stimuli. Phase 1 was practice, Phase 2 was Stim1Part1, Phase 3 was Stim1Part 2, Phase 4 was Practice, Phase 5 was Stim2Part1, Phase 6 was Stim2Part2, and Phase 7 was Stim1Return. Bolded numbers are the critical cross-group comparisons, which are reliable only for the first test phase for the more difficult shape stimuli, BF_incl_ = 3.44.

## Data Availability

Experimental materials, data, and analysis codes can be viewed at an anonymized OSF link: https://osf.io/g2s5h/?view_only=e5dffc06f2cf442081ed97945abf500a.

## Supplementary Materials

The following supporting information can be downloaded at: https://www.mdpi.com/article/10.3390/jintelligence11030056/s1, Figure S1: The mean reaction time in each phase (x-axis) of each group; Figure S2: The bias in each test phase (x-axis) for both groups; Table S1: Statistical results of the frequentist and Bayesian ANOVA for reaction time; Table S2: Three methods for calculating the Bayes factor for reaction time; Table S3: Statistical results of the frequentist and Bayesian ANOVA for bias; Table S4: Three methods of calculating the Bayes factor for bias.
