# Supplementary material for: Generalization of Skill for a Working Memory Recognition Procedure in Children: The Benefit of Starting with Easy Materials
Source: J Intell. 2023 Mar 17;11(3):56. doi: 10.3390/jintelligence11030056 (PMC10057205; doi:10.3390/jintelligence11030056)
Supplement: Supplementary file 1 [file jintelligence-11-00056-s001.zip › jintelligence-2228649-supplementary.pdf]

Generalization of Skill for a Working Memory Recognition Procedure in Children:

The Benefit of Starting with Easy Materials

Supplementary Material

Reaction Time Results

Figure S1 shows the trend of the mean reaction time for five test phases for two groups. The figure indicates that the reaction time of Group 1 was faster than that of Group 2 for each test phase. The two groups had similar trends averaged across trials of five test phases. The mean response time of both groups decreased in the third test phase compared with the second phase. Then the mean reaction time increased in both groups (Phases 5 to 7) when they started to memorize the second set of stimuli.

Figure S1

The mean reaction time in each phase (x-axis) of each group.

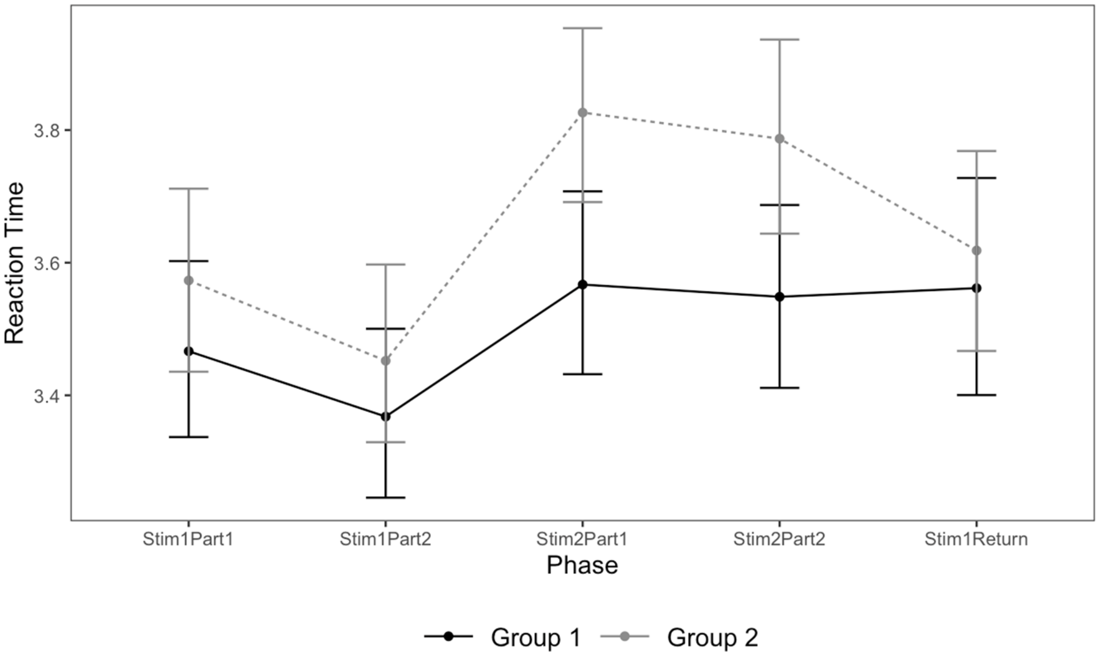

Note. Black line: Group 1 started with the orientation stimuli. Grey line: Group 2 started with the shape stimuli. Error bars are the standard errors of the mean.

ANOVA of Reaction Time

An ANOVA of the reaction time was conducted with one within-participant factor (phase, with five test phases) and one between-participants factor (group, 1 or 2). We examined seven models by comparing them with a null hypothesis model that did not include group, phase, or their interaction. The models, summarized in Table S1, included those with all combinations of the group and phase variables. On the basis of the table, we used the three different methods that were used for proportion correct to calculate the BF_{incl} (the Bayes Factor for including an effect) for phase, group, and their interaction (Table S2). In summary, the best model of the present study was Model 2, which only included the group effect but not the phase or interaction effects.

Table S1

Statistical results of the frequentist and Bayesian ANOVA for reaction time.

Model	Factors	Predictor	*df*		F	Cohen's f	*p*	BF_{incl}
1	Phase	Phase	4	276	2.99	.065	**<.05**	.8317
2	Group	Group	1	6920	10.94	.040	**<.001**	6.3286
3	Phase + group	Phase	4	276	2.99	.065	**<.05**	5.1334
		Group	1	64	.91	.063	.34	
4	Interaction	Interaction	8	272	1.70	.070	.10	.0005
5	Phase + interaction	Phase	4	272	2.97	.065	**<.05**	.0004
		Interaction	4	272	.44	.025	.78	
6	Group + interaction	Group	1	60	.53	.047	.47	.0030
		Interaction	8	272	1.70	.070	.10	
7	Phase + group + interaction	Phase	4	272	2.97	.065	**<.05**	.0025
		Group	1	60	.93	.063	.34	
		Interaction	4	272	.44	.025	.78	

Table S2
Three methods for calculating the Bayes factor for reaction time.

Factor	Method	Formula	BF_{incl}
Phase	1	Phase / null model	.83
	2	(Phase + group) / group	.81
	3	All effects / (group + interaction)	.83
Group	1	Group / null model	6.33
	2	(Group + interaction) / interaction	6.00
	3	All effects / (phase + interaction)	6.25
Interaction	1	Interaction / null model	.0005
	2	(Group + interaction) / group	.0005
	3	All effects / (group + phase)	.0005

Analysis of Bias

The patterns of bias are shown in Figure S2. An ANOVA of bias was conducted with one within-participant factor (phase, with five levels) and one between-participants factor (group, 1 or 2). The value of bias was calculated as the proportion of trials in which the participant answered “yes” to the trials in each phase, that is, (trials answered with “yes, in the sequence”)/(total target-present plus target-absent trials). This analysis still excluded trials with reaction times of 3 SD above the mean. We calculated the values of bias for each participant across the five test trials (see Figure S2). Then we compared seven models with the null hypothesis model, as shown in Table S3. Only Models 4 and 5 showed a reliable BF_{incl} above 3.

Table S3
Statistical results of the frequentist and Bayesian ANOVA for bias.

Model	Factors	Predictor	*df*		F	Cohen's f	*p*	BF_{incl} of Model
1	Phase	Phase	4	276	4.14	.245	**<.001**	.27
2	Group	Group	1	348	1.66	.069	.20	.26
3	Phase + group	Phase	4	276	4.14	.245	**<.001**	.07
		Group	1	68	.60	.105	.44	
4	Interaction	Interaction	8	272	6.45	.435	**<.001**	11.27
5	Phase + interaction	Phase	4	272	4.58	.260	**<.01**	3.47
		Interaction	4	272	8.32	.349	**<.001**	
6	Group + interaction	Group	1	68	.60	.112	.44	2.99
		Interaction	8	272	6.45	.435	**<.001**	
7	Phase + group + interaction	Phase	4	272	4.58	.260	**<.01**	.97
		Group	1	68	.60	.112	.44	
		Interaction	4	272	8.32	.349	**<.001**	

Note. The BF_{incl} of each model was in comparison with the null model.

On the basis of Table S3, we also used three different methods to calculate the BF for interaction, phase, and group effects on bias (Table S4). The results were similar for these three methods. Overall, the best model of the present study was Model 4, which only included the interaction but not the phase and group variables. As shown in Figure S2, this interaction was best described as a different bias for the easier orientation stimuli versus the harder shape stimuli.

Table S4
Three methods of calculating the Bayes factor for bias.

Factor	Method	Formula	BF_{incl}
Phase	1	Phase / null model	.27
	2	(Phase + group) / group	.27
	3	All effects / (group + interaction)	.32
Group	1	Group / null model	.26
	2	(Group + interaction) / interaction	.27
	3	All effects / (phase + interaction)	.28
Interaction	1	Interaction / null model	11.28
	2	(Group + interaction) / group	11.42
	3	All effects / (group + phase)	13.53

Figure S2

The bias in each test phase (x-axis) for both groups.

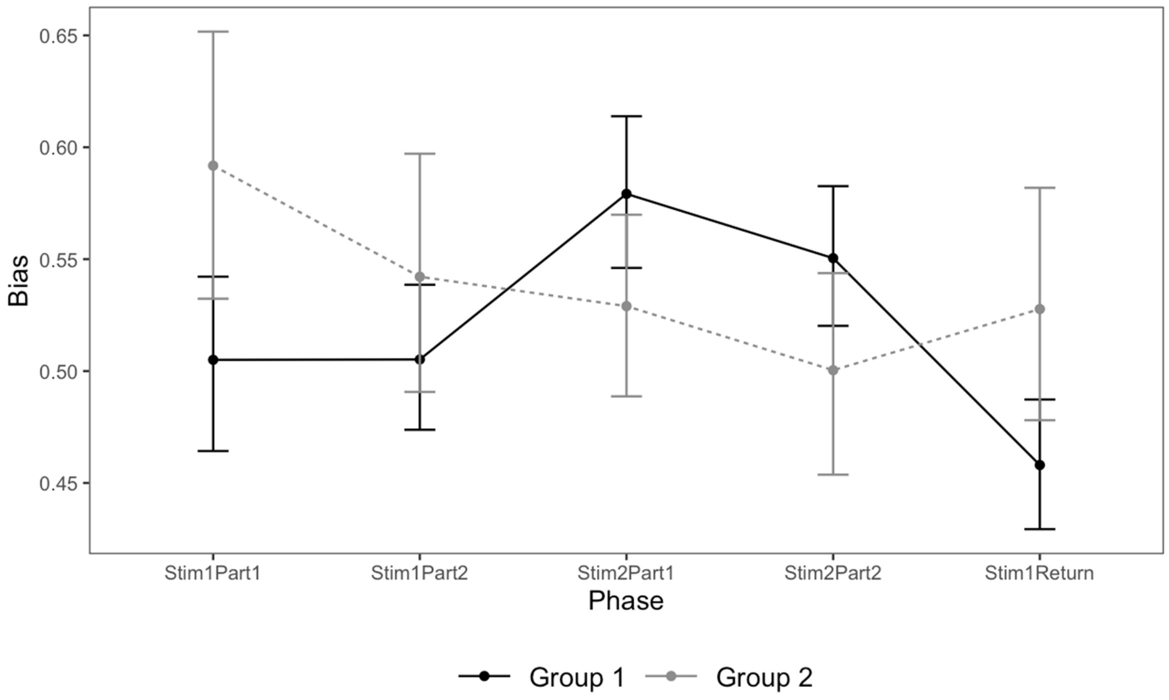

Note. Black line: Group 1 started with the orientation stimuli. Grey line: Group 2 started with the shape stimuli. Error bars are the standard errors of the mean.

Bias Effects on Working Memory

We conducted another Bayesian inference model to explore the effects of the children's bias for each phase and group on working memory training. The results indicated that phase, group, and their interaction were reliable predictors of the children's bias. Because younger children have less attention or fewer resources, they may be prone to rely on a bias that overestimates their abilities (Koriat & Helstrup, 2007). Forsberg et al. (2021) proposed that children may be aware of the items in the focus of attention, but not of the processing factors that decrease their ability to demonstrate a memory of these items. For example, they may not understand that some knowledge of an object does not indicate complete knowledge, or may not consider the role of decay and interference in retaining memory traces. Thus, children may be

more likely to overestimate their abilities compared with adults. In this study, that bias manifested as average bias scores above 0.50.
